# Single-shot intrathecal morphine in bilateral lung transplantation: Early experience and feasibility

**DOI:** 10.1016/j.jhlto.2026.100644

**Published:** 2026-07-23

**Authors:** Harry Pearce, Chinmay Patvardhan

**Affiliations:** Consultant in Anaesthetics and Intensive Care, Department of Anaesthesia and Intensive Care, Royal Papworth Hospital

**Keywords:** Intrathecal morphine, Lung transplantation, Thoracic epidural analgesia, Postoperative analgesia, Extracorporeal membrane oxygenation, Enhanced recovery

## Abstract

Bilateral lung transplantation via clamshell thoracotomy is associated with intense postoperative pain. Thoracic epidural analgesia (TEA) is the institutional standard at our center but carries haemodynamic, catheter-related, and anticoagulation constraints in the early post-transplant period. We describe a case series of 5 bilateral lung transplant recipients in whom single-shot intrathecal morphine (ITM) was used as the primary analgesic technique; the first reported experience of ITM in this setting. Across all cases, effective analgesia was achieved, no patient required TEA rescue, numeric rating scale (NRS) pain scores did not exceed 2 up to 48 hours postoperatively, patient-reported satisfaction was good, and no adverse events attributable to ITM were observed. These findings contribute early safety and efficacy data for ITM in a setting where it has not previously been described.

## Background

Bilateral lung transplantation via clamshell thoracotomy is associated with intense postoperative pain, delayed mobilization, and prolonged intensive care unit (ICU) stay.^1^ Intrathecal morphine (ITM) provides prolonged analgesia and reduced opioid consumption across non-abdominal surgical settings,^2^, ^3^ but its use in lung transplant recipients has not yet been described. We report a case series of 5 bilateral lung transplant recipients who received single-shot ITM.

## Methods

This is an observational case series. Five bilateral lung transplant recipients who received single-shot ITM at Royal Papworth Hospital between November 2025 and March 2026 were identified. ITM was substituted for thoracic epidural analgesia (TEA) as the primary analgesic technique; all other components of the institutional postoperative analgesia protocol remained unchanged. Data were collected from prospectively maintained clinical records. Written informed consent was obtained from all patients.

## Cases

Case 1: A 57-year-old woman with end-stage pulmonary fibrosis (Medical Research Council dyspnea grade 4, 6-minute walk distance 120 m) underwent bilateral orthotopic lung transplantation via clamshell thoracotomy under general anesthesia. She had no other significant comorbidities, was opioid-naive, and had no contraindications to neuraxial anesthesia. Intraoperative extracorporeal membrane oxygenation (ECMO) using a hybrid veno-arterial circuit (via central cannulation) was employed per institutional practice. Following ECMO wean and decannulation, heparin was reversed with protamine. Prior to performing the neuraxial technique, rotational thromboelastometry and standard coagulation studies confirmed fully normalized values.

ITM was selected in preference to our institutional standard of TEA, which is normally placed in the ICU prior to extubation, and was retained as a rescue option should analgesia prove insufficient. Injections were performed under general anesthesia or deep sedation, consistent with established cardiothoracic practice.^2^, ^4^

With confirmed hemostasis, stable hemodynamics, and normalized coagulation tests, a lumbar intrathecal injection of 700 micrograms preservative-free morphine was performed uneventfully at the end of the procedure with the patient under general anesthesia. Prophylactic antiemetics were given, and no neuraxial local anesthetic was used to preserve hemodynamic stability.

The patient was extubated at 6 hours.^5^ She reported no pain for 36 hours, required no intravenous opioid rescue, and mobilized from postoperative day 1. A single oral oxycodone dose was required at 36 hours; she was discharged from the ICU on day 2.

Cases 2 to 5 were 4 bilateral lung transplant recipients, all undergoing clamshell thoracotomy with intraoperative hybrid veno-arterial ECMO. Single-shot ITM was placed either in theater or on arrival to the ICU. Outcomes are summarized in Table 1. Effective analgesia was achieved in all cases and no patient required TEA. Patient-reported satisfaction with analgesia was documented as good in all cases during ICU admission; no patient recorded numeric rating scale pain scores greater than 2 up to 48 hours postoperatively. No adverse events likely or possibly attributable to ITM were observed.^8^

**Table 1:** Outcomes Across All 5 Cases

Case	Type	ITM dose (µg)	Setting	Patient state	Time to extubation (h)	Breakthrough analgesia within 48 h of insertion	TEA rescue	ICU LOS (days)	Adverse events attributable to ITM
1	BLT	700	Theatre	Under GA	6	10 mg oral oxycodone at 36 hours	No	2	None
2	BLT	600	Theatre	Under GA	8	None before 48 hours	No	2	None
3	BLT	700	ICU	Sedated/intubated	11	None before 48 hours	No	2	None
4	BLT	500	ICU	Sedated/intubated	46*	None before 48 hours	No	4	None
5	BLT	500	Theatre	Under GA	6	10 mg oral morphine at 28 hours	No	2	None

Summary of outcomes across all 5 cases. BLT, bilateral lung transplant; GA, general anesthesia; ICU LOS, intensive care unit length of stay; ITM, intrathecal morphine; TEA, thoracic epidural analgesia. *Extubation delayed while evaluating postoperative bleeding.

## Discussion

Compared with TEA, ITM avoids hemodynamic compromise from local anesthetic infusion, the anticoagulation constraints imposed by an indwelling catheter (relevant in the context of ECMO-associated or pathophysiological coagulopathy), and the need for repeat neuraxial procedures in the event of catheter failure.^9^, ^10^ A single-shot technique requires no ongoing management and preserves TEA as a rescue option should analgesia prove insufficient.

Patient selection was at anesthetist discretion, using the following criteria: no neuraxial contraindications, normalized coagulation, and an anticipated uncomplicated postoperative course. As sicker recipients have worse pain trajectories,^7^ this may have favored those at lower analgesic risk, and the series cannot distinguish ITM's effect from the natural postoperative trajectory. This is the most important limitation of the series.

The finite duration of ITM is its key practical limitation relative to continuous TEA. However, postoperative pain is typically most intense in the early hours after surgery, and ITM’s duration of action coincides with this window, when respiratory function and early mobilization are most critical for graft recovery. Notably, in Case 4, extubation was delayed while evaluating postoperative bleeding; ventilatory parameters were met throughout, and analgesic adequacy after extubation remained satisfactory on a standard oral regimen. This is consistent with the principle of matching ITM’s peak effect to the period of most intense postoperative pain, with conventional multimodal analgesia sufficient thereafter.

Dosing varied between cases at the treating anesthetist's discretion. Although 5 μg/kg is considered optimal for open thoracotomy^6^ and no late respiratory depression reported below 500 micrograms^2^, 3 cases received doses above this threshold (600-700 micrograms), reflecting the treating anesthetist’s assessment that the clamshell incision presents a greater pain stimulus than the procedures in the current evidence base. The higher side-effect risk was accepted given the availability of close ICU monitoring; no adverse effects attributable to ITM were observed across any case.

## Conclusion

The primary aim of this series was to demonstrate feasibility: as ITM has not previously been described in lung transplantation, we present these cases to establish that the technique is deliverable in this setting and to report early safety signals.

Our experience raises questions that prospective evaluation could address. Specific areas include: enrollment of higher-complexity recipients; protocol-defined dosing to optimize the efficacy-safety balance; systematic pain score collection to characterize analgesic duration; and evaluation of analgesia beyond the 48-hour window as ITM’s effect wanes.

## Conflicts of Interest statement

The authors declare that they have no known competing financial interests or personal relationships that could have appeared to influence the work reported in this paper.
